# Plasma Trans Fatty Acid Levels, Cardiovascular Risk Factors and Lifestyle: Results from the Akershus Cardiac Examination 1950 Study

**DOI:** 10.3390/nu12051419

**Published:** 2020-05-14

**Authors:** Anupam Chandra, Magnus Nakrem Lyngbakken, Ivar Anders Eide, Helge Røsjø, Thea Vigen, Håkon Ihle-Hansen, Eivind Bjørkan Orstad, Ole Morten Rønning, Trygve Berge, Erik Berg Schmidt, Arnljot Tveit, Torbjørn Omland, My Svensson

**Affiliations:** 1 Department of Renal Medicine, Division of Medicine, Akershus University Hospital, 1478 Lørenskog, Norway; Ivar.anders.eide@gmail.com (I.A.E.); m.h.s.svensson@medisin.uio.no (M.S.); 2 Institute of Clinical Medicine, University of Oslo, 0315 Oslo, Norway; magnus.nakrem.lyngbakken@gmail.com (M.N.L.); helge.rosjo@medisin.uio.no (H.R.); theavigen@gmail.com (T.V.); o.m.ronning@medisin.uio.no (O.M.R.); arnljot.tveit@vestreviken.no (A.T.); torbjorn.omland@medisin.uio.no (T.O.); 3 Division of Medicine, Akershus University Hospital, 1478 Lørenskog, Norway; Eivind.Bjorkan.Orstad@ahus.no; 4 Division of Research and Innovation, Akershus University Hospital, 1478 Lørenskog, Norway; 5 Department of Medical Research, Bærum Hospital, Vestre Viken Hospital Trust, 3004 Drammen, Norway; haaihl@vestreviken.no (H.I.-H.); tryber@vestreviken.no (T.B.); 6 Department of Cardiology, Aalborg University Hospital, 9100 Aalborg, Denmark; ebs@rn.dk

**Keywords:** industrial trans fatty acids, ruminant trans fatty acids, cardiovascular health, legislative bans, cardiovascular risk factors

## Abstract

Intake of industrially produced trans fatty acids (iTFAs) has previously been associated with dyslipidemia, insulin resistance, hypertension and inflammation, as well as increased cardiovascular (CV) morbidity and mortality. iTFA intake declined in Norway after the introduction of legislative bans against iTFA consumption. However, the relationship between the current iTFA intake and CV health is unclear. The aim of the present study was to investigate the association between current iTFA intake, reflected by plasma iTFA levels, and established CV risk factors. We also examined the associations between plasma ruminant TFA levels and CV risk factors. In this cross-sectional study, we included 3706 participants from a Norwegian general population, born in 1950 and residing in Akershus County, Norway. The statistical method was multivariable linear regression. Plasma iTFA levels were inversely associated with serum triglycerides (*p* < 0.001), fasting plasma glucose (*p* < 0.001), body mass index (*p* < 0.001), systolic and diastolic blood pressure (*p* = 0.001 and *p* = 0.03) and C-reactive protein (*p* = 0.001). Furthermore, high plasma iTFA levels were associated with higher education and less smoking and alcohol consumption. We found that plasma ruminant trans fatty acids (rTFA) levels were favorably associated with CV risk factors. Furthermore, plasma iTFA levels were inversely associated with CV risk factors. However, our results might have been driven by lifestyle factors. Overall, our findings suggest that the current low intake of iTFAs in Norway does not constitute a threat to CV health.

## 1. Introduction

Trans fatty acids (TFAs) are unsaturated fatty acids with at least one double bond in trans configuration [1]. The two major sources of TFAs are partially hydrogenated vegetable oils, found in various industrially prepared foods, and in dairy and meat products from ruminants [1]. Epidemiological studies have shown a strong association between intake of TFAs, predominantly industrial produced TFAs (iTFAs), and risk of cardiovascular (CV) disease [2]. The first major study was published in the early ‘90 s [3], followed by numerous studies consistently reporting harmful influence of iTFAs on CV health [4,5,6,7]. Therefore, the intake of iTFAs was considered a major public health problem, forcing governments around the world to take legislative action to reduce iTFA content in foods [8,9]. In addition, cooperative efforts by food industries, voluntarily excluding iTFA-rich products from their assortment and providing better food labelling for consumers, resulted in a further reduction of iTFA consumption [1]. In 2003, the World Health Organization recommended to limit the intake of iTFAs to <1% of overall energy consumption [10].

In Norway, the total TFA intake was approximately 5% of overall energy consumption during the late ‘50 s, gradually decreasing over the next decades, mainly due to reduction in the use of margarines [11]. A large Norwegian cohort study, including participants between 1974 and 1988, reported a positive association between consumption of iTFAs and CV death [12]. The mean intake of iTFAs was 0.9–1.6% of energy consumption during the study period. Since then, the impact of iTFAs on CV health has not been evaluated in a Norwegian general population. 

There is an increased risk of CV disease even at low levels of iTFAs, and an intake of <0.5% of overall energy consumption might be necessary to avoid adverse effects [2]. Whether the current iTFA consumption correlates with established CV risk factors is not known. Moreover, as a consequence of reduced iTFA consumption, ruminant TFAs (rTFAs) are now the major TFAs in the daily Nordic diet [13], but the relationship between rTFAs and CV health has been less studied. Accordingly, in this study, the aim was to examine the associations between the current intake of iTFAs, reflected by plasma phospholipid levels [14], and CV risk factors in a middle-aged Norwegian community-acquired cohort. In addition, we examined the association between current rTFA intake and CV risk factors. 

## 2. Materials and Methods 

### 2.1. Study Cohort

**Figure 1:**
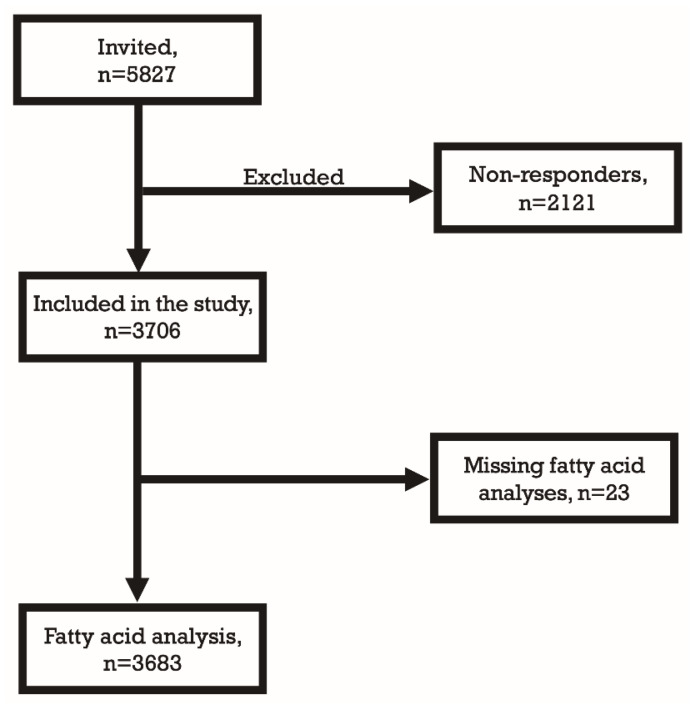
Flowchart for inclusion of study participants.

The Akershus Cardiac Examination (ACE) 1950 Study is a collaborative project between Akershus University Hospital and Bærum Hospital, Vestre Viken Hospital Trust, Norway. It is a population-based cohort study aimed to examine CV health of individuals born in 1950 and residing in Akershus County, Norway. The study design has previously been presented in detail [15]. From a total of 5827 eligible individuals invited for participation, 3706 (64%) were enrolled in the study (Figure 1). The remaining 2121 (36%) declined participation or did not respond.

### 2.2. Study Variables 

Participants were interviewed regarding medical history, diet, education, physical activity and smoking habits. Cerebrovascular and coronary artery disease were self-reported. Cerebrovascular disease was classified as history of stroke or cerebral hemorrhage, while coronary artery disease was defined as history of acute myocardial infarction or having undergone percutaneous coronary intervention or coronary artery bypass graft operation. Dietary data were self-reported and collected using a previously validated questionnaire [16], where the participant indicated type of dietary item and frequency of intake. Higher education was defined as >12 years of formal education, such as college or university education at any level. High physical activity was self-reported and defined as vigorous-intensity exercise for more than 30 min, at least twice weekly [17]. Smoking habits were recorded as either current smoker or previous/non-smoker. Alcohol consumption was recorded as intake of alcohol two times or more per week. We defined hypertension as current use of any anti-hypertensive medication, or as mean systolic blood pressure ≥140 mmHg or mean diastolic blood pressure ≥90 mmHg obtained from three measurements in sitting position after 10 min rest [18]. The World Health Organization criterion (body mass index [BMI, kg/m^2^] ≥30) was used to define obesity [19]. 

Blood samples were drawn after fasting overnight, immediately frozen and stored at −80 °C. We defined hypercholesterolemia as current use of any lipid-lowering agent or total serum cholesterol ≥6.2 mmol/L or low-density lipoprotein (LDL) cholesterol ≥4.1 mmol/L [20]. Diabetes mellitus (DM) was defined as current use of any glucose-lowering medication, self-reported DM or both fasting plasma glucose (FPG) ≥7.0 mmol/L and glycated hemoglobin (HbA1c) ≥6.5% [21]. The Chronic Kidney Disease Epidemiology Collaboration equation [22] was used to calculate estimated glomerular filtration rate (eGFR), and chronic kidney disease stages 3–5 were defined as eGFR <60 mL/min/1.73 m^2^. C-reactive protein (CRP) was measured using standard assay. Carotid intima-media thickness (cIMT) of the left and right common carotid arteries were assessed by ultrasound examination, and mean cIMT was obtained as previously described [23].

Samples of frozen plasma were sent to The Lipid Research Center, Aalborg University Hospital for phospholipid fatty acid determination by gas chromatography. We used modified Folch and Burge methods to extract total lipids from serum and to isolate the phospholipid fraction from other lipids [24]. A Varian 3900 gas chromatograph (Varian, Middleburg, The Netherlands) with 60 m × 0.25 mm capillary columns was used for fatty acids analysis. Individual fatty acids were identified and quantified as weight percentage (wt%) of total plasma phospholipid fatty acids. Fatty acid analysis was not performed in 23 participants because of inadequate plasma volume (Figure 1). We defined vaccenic acid and trans-conjugated-linoleic acid as rTFAs [25]. All other TFAs, such as trans-elaidic acid, 18:1d6-8t and 18:3ttt were defined as iTFAs. 

### 2.3. Statistical Analysis 

Quartiles of plasma iTFA levels were used for the presentation of clinical and demographic data. Results are given as mean values (standard deviations) for normally distributed data, median values (inter-quartile ranges) for skewed data (serum triglycerides, FPG, HbA1c and CRP) and percentages for categorical data. Differences between quartiles of plasma iTFA levels were examined by using ANOVA for normally distributed continuous variables, the Kruskal-Wallis test for skewed variables and Chi square test for binary data.

We evaluated associations between plasma iTFA and rTFA levels and CV risk factors with multivariable linear regression, after testing assumptions for linearity. Separate models were created for each CV risk factor to examine associations between industrial and ruminant TFAs and various CV risk factors individually. Adjustments were made with predefined candidate variables. The simultaneous entry method was used for all models. To obtain normal distribution, skewed variables such as triglycerides, FPG, HbA1c and CRP were logarithmically transformed. Thus, for these variables, the presented unstandardized regression coefficients (Unstd. β-coeff.) and corresponding 95% confidence intervals (CI) represent the anti-logarithm of acquired results. Unstd. β-coeff. with corresponding 95% CI, standardized regression coefficients (Std. β-coeff.), *p*-values and explained variance (R^2^) for the univariable models and fully adjusted final models are presented in the tables. Statistics were preformed using SPSS^®^ version 25.0 (IBM, New York, NY, US) and STATA 16 (StataCorp LP, Texas, TX, USA).

### 2.4. Ethics 

This study was performed according to the Declaration of Helsinki guidelines. Regional Ethics Committee for Medical and Health Research Ethics approved all procedures involving research study participants (September 7^th^, 2011. Reference number 2011/1475). Participants gave written informed consents before final enrolment.

## 3. Results

### 3.1. Demographics 

**Figure 2:**
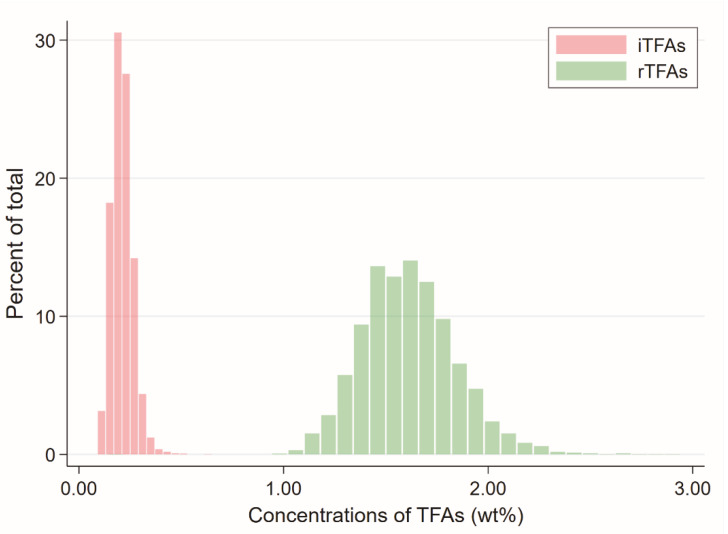
Distribution of industrial and ruminant trans fatty acids measured in weight percentage (wt%) of total plasma phospholipid fatty acids.

Demographics and clinical characteristics of participants stratified according to quartiles of plasma iTFA levels are given in Table 1. Plasma iTFA levels ranged from 0.09 to 0.62 wt%, with a median level of 0.20 wt% (interquartile range 0.13 to 0.27 wt%) (Figure 2). 

There was a higher proportion of females in the upper quartile of plasma iTFA levels. Using the lower quartile of plasma iTFA levels as reference, participants in the upper quartile were more educated, had a lower prevalence of smoking and consumed alcohol less often. Furthermore, participants in the upper quartile of plasma iTFA levels had a lower prevalence of comorbidities, such as hypertension, hypercholesterolemia, coronary artery disease, DM and obesity, and less use of antihypertensive, lipid-lowering and glucose-lowering drugs. 

Demographics and clinical characteristics of participants stratified according to quartiles of plasma rTFA levels are presented in Supplementary Table S1. Plasma rTFA levels ranged from 0.14 to 2.87 wt%, with a median level of 1.60 wt% (interquartile range 1.30 to 1.90 wt%) (Figure 2). Using the lower quartile of plasma rTFA levels as reference, participants in the upper quartile had a higher consumption of margarine and butter, a higher prevalence of smoking and consumed alcohol more often. In addition, they had a lower prevalence of DM, but higher prevalence of coronary artery disease and used more lipid-lowering drugs.

### 3.2. Plasma iTFA Levels and CV Risk Factors 

Unadjusted and multivariable adjusted associations between plasma iTFA levels and CV risk factors are presented in Table 2. High levels of plasma iTFAs were associated with lower serum triglycerides, FPG levels, BMI, systolic and diastolic blood pressure and CRP levels. We found no associations between plasma iTFA levels and serum high-density lipoprotein (HDL) or LDL cholesterol levels, HbA1c, eGFR or cIMT.

### 3.3. Plasma rTFA Levels and CV Risk Factors

Unadjusted and multivariable adjusted associations between plasma rTFA levels and CV risk factors are presented in Table 3. High levels of plasma rTFAs were associated with higher serum HDL and lower serum LDL cholesterol levels, serum triglycerides, FPG levels, HbA1c, BMI, systolic and diastolic blood pressure. We found no associations between plasma rTFA levels and eGFR, cIMT or CRP levels.

## 4. Discussion

The main finding in the present study was that plasma rTFA levels were favorably associated with CV risk factors. Furthermore, plasma iTFA levels were inversely associated with several risk factors for CV disease, such as serum triglycerides, FPG, BMI, systolic and diastolic blood pressure and CRP levels. These results are in apparent contrast to the established harmful effects of iTFA on CV health, and might have been confounded by lifestyle related factors. Our findings suggest that the current low intake of iTFAs in Norway does not constitute a threat to CV health. 

### 4.1. Plasma iTFA Levels and CV Risk Factors

iTFAs are suggested to promote dyslipidemia, inflammation and endothelial dysfunction [26,27]. These adverse effects of iTFAs have been related to the trans double bonds, changing their configuration and chemical properties compared with their cis-isomers [28]. 

iTFAs have been used in the food industry since the 1950 s [29]. They were produced by partial hydrogenation of vegetable and fish oils, turning them into semi-solid fats that were cheap to produce, and with desirable physical properties like long shelf life and temperature stability [1]. Consequently, iTFAs were widely used in products like margarines, bakery products, crackers and deep-fried food [29]. The negative effects of iTFAs first became apparent from studies performed during the last few decades [2,3,30]. In a meta-analysis of four prospective studies, a 2% increase in energy intake from iTFAs was associated with a 23% increase in incident of CV disease [2]. From a nutritional standpoint, iTFAs were potentially harmful, and had no apparent health benefits. Consequently, several countries introduced legislation mandating the reduction of iTFA concentrations in foods [31], with Denmark being the first country to do so in 2003 [8]. Although the Norwegian iTFA-legislation was not passed before 2014 [31], the iTFA consumption was reduced shortly after the Danish legislation, mostly due to societal pressure and efforts by Regulatory Authorities and the Norwegian food industry [11]. In 2012, the dietary intake of total TFAs was <1% of total energy consumption in Norway [32].

We have previously shown a reduction in median plasma iTFA levels from 0.29 wt% in years 1999–2004 to 0.20 wt% in years 2005–2011 in a Norwegian population of kidney transplant recipients [33]. Similarly, the median plasma iTFA level was 0.20 wt% in the present study, suggesting a persistent low iTFA consumption in the Norwegian population. iTFAs have previously been associated with hypertriglyceridemia, insulin resistance, adiposity, hypertension and inflammation [26,34,35,36,37]. In contrast, we found inverse associations between plasma iTFA levels and serum triglycerides, FPG, BMI, systolic and diastolic blood pressure and CRP levels. In addition, the highest levels of plasma iTFAs were found in participants with high education, a healthy lifestyle with less fast food consumption, and lower prevalence of comorbidities. 

These findings were unexpected and seemingly paradoxical. The detrimental effects of iTFAs are scientifically established, thus, the biological plausibility of high iTFA intake being beneficial to CV health seems highly unlikely. In Norway, potential sources of iTFAs might be consumption of imported food products that still contain high levels of iTFAs [38], or traveling to destinations with less restrictive or no iTFA-bans. However, we do not have data to support these assumptions, and the reasons for these findings remain to be elucidated. It is worth noticing that our results are characterized by small regression coefficients, signaling no associations between plasma iTFA levels and CV risk factors, rather than an inverse relationship. Our findings suggest that the low levels of plasma iTFAs observed in this cohort are perhaps no longer of clinical relevance, and that the inverse associations between plasma iTFA levels and CV risk factors might have been confounded by factors related to healthy lifestyle, not fully adjusted for in the regression analyses. 

### 4.2. Plasma rTFA Levels and CV Risk Factors

Reduction in iTFA consumption has not affected the rTFA intake, at least not in Scandinavian countries, where the intake has been relative constant since the 1990 s [39,40]. Epidemiological studies have reported a nonsignificant trend towards an inverse association between rTFA intake and risk of CV disease [3,5]. Reasons for these findings are unclear, but might be related to different biological properties of rTFAs compared to iTFAs, or the presence of other substances in dairy and meat products that might be protective against CV disease [1]. 

A moderate intake of rTFAs has resulted in a favorable lipid profile, with higher serum HDL and lower serum LDL cholesterol levels, in clinical trials [41]. However, a high intake of rTFAs increase serum LDL cholesterol levels [41]. In this study, plasma rTFA levels were positively associated with serum HDL cholesterol levels and inversely associated with serum LDL cholesterol levels and serum triglycerides. These findings indicate favorable relationships between high rTFA intake and blood lipids. However, participants in the upper quartile of plasma rTFA levels used more lipid-lowering drugs, which might have confounded the association between plasma rTFA levels and serum LDL cholesterol levels.

We also found inverse associations between plasma rTFA levels and FPG levels and HbA1c. In addition, participants with the highest plasma rTFA levels had a lower prevalence of DM, a finding in line with previous epidemiological studies reporting an inverse association between rTFA intake and incident DM [42,43]. Suggested mechanisms for this observation are rTFA-mediated increased insulin sensitivity and skeletal muscle glucose-uptake [42]. However, in clinical trials, diet rich in vaccenic acid, the major rTFA, has not shown any effect on fasting insulin or glucose [44]. Thus, the relationship between rTFA intake and glucose metabolism remains unclear. Finally, we found inverse associations between plasma rTFA levels and BMI and blood pressure. Epidemiological data on rTFA intake and BMI are inconclusive [45,46], and no effect of rTFAs consumption on blood pressure has been seen in clinical trials [47]. Interestingly, participants in the upper quartile of plasma rTFA levels had an overall unhealthier lifestyle with higher consumption of margarine and butter, higher prevalence of smoking and alcohol consumption. This might have contributed to the higher prevalence of coronary artery disease among these participants.

We found overall favorable associations between plasma rTFA levels and CV risk factors. However, similar to plasma iTFAs levels, the demonstrated associations to CV risk factors were weak, although statistically significant. 

### 4.3. iTFA Legislation and Global Aspects

In this paper we briefly summarize the iTFA story in Norway, with a snapshot of the current status, characterized by a low iTFA intake that is unlikely to increase in the future. As pointed out by Brouwer et al. in the article “Trans fatty acids and cardiovascular health: research completed?”, due to well documented detrimental effects the use of iTFAs is nearly eliminated in many countries and unlikely to be brought back into the food industry [48]. Unfortunately, there are still large regions worldwide, such as North America and the Middle East, where the consumption of iTFAs by far exceeds the recommendations given by the World Health Organization [49,50], reasons for this being ineffective strategies for voluntary iTFA reduction in foods and the lack of legislative iTFA bans [31]. In a recent publication, Wilczek et al. expresses the urgent need for such legislative action due to alarmingly high iTFA consumption in Eastern and South-Eastern Europe [29]. It is estimated that the Danish policy against iTFA reduced CV disease mortality by 14.2 deaths per 100,000-person years on average in the period 2004–2006 [51]. We hope that such findings, as well as our own, can inspire governments in countries with a high iTFA intake to implement measures that can effectively reduce the iTFA consumption. 

### 4.4. Strengths and Limitations 

The present study has several strengths, including a well-described large study population with little missing data. All the study participants were born in 1950, removing age as possible confounding element. In addition, plasma phospholipid TFA levels were determined by gas chromatography, giving an estimate of TFA intake [14]. One of the main limitations is the cross-sectional study design. We do not have dietary data to determine the absolute iTFA or rTFA intake. Reverse causality bias cannot be ruled out. Furthermore, the influence of residual confounding on the associations between plasma iTFA and rTFA levels and CV risk factors cannot be excluded, despite adjustments for covariates. Finally, our result might have limited generalizability to other age groups and ethnicities. 

## 5. Conclusions

In this cross-sectional study of Norwegian individuals born in 1950, plasma rTFA and iTFA levels were favorably associated with CV risk factors. Plasma iTFA levels were low, and the weak associations to CV risk factors might have been confounded by factors related to a healthy lifestyle, such as less smoking and alcohol consumption and a higher educational level. Overall, our findings suggest that the current low intake of iTFAs in Norway no longer poses a threat to CV health. 

## Figures and Tables

**Table 1:** Characteristics of participants according to quartiles of industrial trans fatty acid levels.

	All Participants	Quartile 1	Quartile 2	Quartile 3	Quartile 4	*p* for Trend	*p* for Q1 vs. Q4
Industrial trans fatty acid level, wt%	0.09–0.62	≤0.17	0.18–0.20	0.21–0.24	≥0.25		
Number of participants	3683	1025	886	893	879		
Age, years	63.9 (0.6)	63.8 (0.6)	63.9 (0.7)	63.9 (0.7)	64.0 (0.7)	<0.001	<0.001
Sex (Male), *%*	51.3 (*n* = 1890)	59.1 (*n* = 606)	54.1 (*n* = 479)	48.7 (*n* = 435)	42.1 (*n* = 370)	<0.001	<0.001
Weekly intake of milk, 1 glass or more, *%*	56.5 (*n* = 2042)	55.4 (*n* = 560)	55.2 (*n* = 476)	58.0 (*n* = 508)	57.6 (*n* = 498)	0.52	0.34
Use butter or margarine in cooking, *%*	50.4 (*n* = 1782)	50.1 (*n* = 496)	51.8 (*n* = 441)	50.6 (*n* = 431)	48.9 (*n* = 414)	0.68	0.61
Use oil in cooking, *%*	48.2 (*n* = 1706)	48.6 (*n* = 481)	46.1 (*n* = 392)	48.3 (*n* = 411)	49.8 (*n* = 422)	0.47	0.60
Weekly intake of fast food, once or more, *%*	18.1 (*n* = 651)	20.7 (*n* = 208)	18.2 (*n* = 157)	18.9 (*n* = 165)	14.0 (*n* = 121)	0.002	<0.001
Current smoker, *%*	14.5 (*n* = 530)	16.7 (*n* = 171)	14.9 (*n* = 131)	13.1 (*n* = 115)	13.0 (*n* = 113)	0.06	0.02
Alcohol consumption, (≥2 times weekly), *%*	47.4 (*n* = 1739)	55.4 (*n* = 568)	50.0 (*n* = 440)	43.6 (*n* = 388)	39.2 (*n* = 343)	<0.001	<0.001
Alcohol consumption, >5 units at same occasion, %	46.6 (*n* = 1701)	56.4 (*n* = 576)	49.1 (431)	43.5 (*n* = 383)	35.9 (*n* = 311)	<0.001	<0.001
Physical activity (≥2 times weekly), *%*	61.7 (*n* = 2240)	59.1 (*n* = 600)	61.5 (*n* = 536)	63.4 (*n* = 554)	63.4 (*n* = 550)	0.17	0.06
Higher education, *%*	46.5 (*n* = 1708)	39.0 (*n* = 399)	48.6 (*n* = 429)	48.8 (*n* = 435)	50.7 (*n* = 445)	<0.001	<0.001
Hypertension, *%*	62.0 (*n* = 2282)	70.5 (*n* = 723)	60.2 (*n* = 549)	59.6 (*n* = 532)	54.4 (*n* = 478)	<0.001	<0.001
Hypercholesterolemia, *%*	52.6 (*n* = 1931)	57.3 (*n* = 584)	50.5 (*n* = 446)	54.4 (*n* = 485)	47.4 (*n* = 416)	<0.001	<0.001
Cerebrovascular disease, *%*	3.7 (*n* = 138)	3.2 (*n* = 33)	3.3 (*n* = 29)	4.7 (*n* = 42)	3.9 (*n* = 34)	0.30	0.44
Coronary artery disease, *%*	7.0 (*n* = 259)	9.7 (*n* = 99)	6.9 (*n* = 61)	6.2 (*n* = 55)	5.0 (*n* = 44)	0.001	<0.001
Diabetes mellitus, *%*	8.5 (*n* = 313)	12.9 (*n* = 132)	7.8 (*n* = 69)	6.7 (*n* = 60)	5.9 (*n* = 52)	<0.001	<0.001
Obesity (BMI ≥ 30), *%*	22.6 (*n* = 831)	27.7 (*n* = 284)	21.3 (*n* = 189)	23.2 (*n* = 207)	17.2 (*n* = 151)	<0.001	<0.001
CKD stages 3–5 (eGFR <60 mL/min × 1.73 m^2^), *%*	3.9 (*n* = 142)	3.9 (*n* = 40)	2.6 (*n* = 23)	3.7 (*n* = 33)	5.3 (*n* = 46)	0.04	0.16
Medication, *%*							
Diuretics	3.1	5.0	2.3	2.5	2.3	0.001	<0.001
Beta blockers	13.4	18.6	13.7	10.8	9.6	<0.001	<0.001
Calcium-channel blockers	8.1	10.7	9.0	6.7	5.7	<0.001	<0.001
ACEi or ARB	26.9	34.2	26.5	24.3	21.5	<0.001	<0.001
Lipid-lowering drugs	26.1	31.4	27.3	24.3	20.7	<0.001	<0.001
Glucose-lowering drugs	5.4	7.6	5.4	4.6	3.6	<0.001	<0.001
Systolic blood pressure, mmHg	138 (19)	140 (19)	138 (19)	137 (19)	136 (18)	<0.001	<0.001
Diastolic blood pressure, mmHg	77 (10)	78 (10)	77 (10)	77 (10)	76 (10)	<0.001	<0.001
HDL cholesterol, mmol/L	1.5 (0.5)	1.5 (0.5)	1.5 (0.5)	1.6 (0.5)	1.6 (0.5)	<0.001	<0.001
LDL cholesterol, mmol/L	3.3 (1.0)	3.3 (1.0)	3.2 (1.0)	3.4 (1.0)	3.3 (1.0)	0.01	0.22
Triglycerides, mmol/L	1.2 (0.4–2–0)	1.4 (0.5–2.3)	1.2 (0.3–2.1)	1.1 (0.4–1.8)	1.0 (0.3–1.7)	<0.001	<0.001
FPG, mmol/L	5.3 (4.5–6.1)	5.4 (4.5–6.3)	5.3 (4.5–6.1)	5.2 (4.4–6.0)	5.2 (4.4–6.0)	<0.001	<0.001
HbA1c, %	5.7 (5.3–6.1)	5.7 (5.2–6.2)	5.7 (5.3–6.1)	5.7 (5.3–6.1)	5.7 (5.3–6.1)	0.004	0.002
Body mass index (BMI), kg/m^2^	27.1 (4.4)	28.0 (4.4)	27.2 (4.3)	27.0 (4.5)	26.2 (4.3)	<0.001	<0.001
eGFR, ml/min × 1.73m^2^	83 (11.9)	83 (12.2)	84 (11.4)	83 (11.7)	83 (12.4)	0.18	0.14
cIMT, mm	0.73 (0.1)	0.73 (0.1)	0.73 (0.1)	0.73 (0.1)	0.72 (0.1)	0.52	0.17
CRP, mg/L	1.5 (1.4–1.6)	1.5 (1.4–1.6)	1.5 (1.4–1.6)	1.5 (1.4–1.6)	1.5 (1.4–1.6)	-	-
Marine n-3 PUFAs, wt%	8.1 (2.6)	8.2 (2.8)	8.2 (2.7)	8.0 (2.5)	7.9 (2.5)	0.08	0.03
LA, wt%	20.8 (3.0)	20.1 (3.0)	20.6 (2.9)	21.1 (2.8)	21.4 (2.9)	<0.001	<0.001
AA, wt%	9.2 (2.0)	9.5 (2.1)	9.3 (2.1)	9.2 (2.0)	8.9 (2.0)	<0.001	<0.001
SFA, wt%	42.4 (0.9)	42.8 (0.8)	42.5 (0.8)	42.3 (0.7)	42.0 (0.9)	<0.001	<0.001
MUFA, wt%	10.3 (1.4)	10.3 (1.5)	10.3 (1.4)	10.2 (1.4)	10.3 (1.3)	0.17	0.84

Results are given as mean values (standard deviation) for continuous data, median values (inter-quartile ranges) for skewed data and percentages for categorical data. Differences between the quartiles were evaluated using Chi square for binary data, the Kruskal-Wallis test for triglycerides, FPG, HbA1c and CRP, and ANOVA for other continuous data. Fatty acids are given as weight percentage (wt%) of total plasma phospholipid fatty acids. Abbreviations: BMI: Body mass index. CKD: Chronic kidney disease. eGFR: Estimated glomerular filtration rate (CKD-EPI formula). ACEi: Angiotensin converting enzyme inhibitor. ARB: Angiotensin receptor blocker. HDL: High density lipoprotein. LDL: Low density lipoproteins. FPG: Fasting plasma glucose. HbA1c: Hemoglobin A1c. cIMT: Carotid intima-media thickness. CRP: C-reactive protein. PUFA: Polyunsaturated fatty acids. LA: Linoleic acid. AA: Arachidonic acid. SFA: Saturated fatty acids. MUFA: Monounsaturated fatty acids.

**Table 2:** Associations between plasma industrial trans fatty acid levels and cardiovascular risk factors.

**Univariable Linear Regression Analysis**					
**Cardiovascular Risk Factors**	***n***	**Unstd. β-coeff. (95% CI)**	**Std. β-coeff.**	***p***	**R^2^**
HDL cholesterol, mmol/L	3680	1.02 (0.72, 1.32)	0.11	<0.001	0.01
LDL cholesterol, mmol/L	3657	0.55 (−0.05, 1.15)	0.03	0.07	0.001
Triglycerides, mmol/L	3680	−7.89 (−10.57, −5.89)	−0.22	<0.001	0.05
FPG, mmol/L	3675	−1.62 (−1.81, −1.47)	−0.15	<0.001	0.02
HbA1c, %	3669	−1.13 (−1.21, −1.06)	−0.07	<0.001	0.004
BMI, kg/m^2^	3683	−13.50 (−16.23, −10.78)	−0.16	<0.001	0.03
SBP	3679	−30.09 (−41.77, −18.42)	−0.08	<0.001	0.01
DBP	3679	−16.45 (−22.70, −10.20)	−0.09	<0.001	0.01
eGFR, mL/min × 1.73m^2^	3664	−5.43 (−12.90, 2.04)	−0.02	0.15	0.00
cIMT, mm	3661	−0.07 (−0.14, 0.004)	−0.03	0.07	0.001
CRP, mg/L	3669	−3.26 (−4.90, −2.14)	−0.09	<0.001	0.01
**Multivariable Linear Regression Analysis**					
**Cardiovascular Risk Factors**	***n***	**Unstd. β-coeff. (95% CI)**	**Std. β-coeff.**	***p***	**R^2^**
HDL cholesterol, mmol/L ^a^	3640	0.20 (−0.06, 0.46)	0.02	0.14	0.30
LDL cholesterol, mmol/L ^b^	3617	−0.44 (−0.97, 0.10)	−0.02	0.11	0.27
Triglycerides, mmol/L ^c^	3640	−4.19 (−5.52, −3.17)	−0.15	<0.001	0.21
FPG, mmol/L ^d^	3636	−1.19 (−1.29, −1.09)	−0.05	<0.001	0.39
HbA1c, % ^e^	3630	−1.01 (−1.06, 1.04)	−0.004	0.77	0.38
BMI, kg/m^2 f^	3612	−11.22 (−13.90, −8.53)	−0.13	<0.001	0.12
SBP ^g^	3640	−20.62 (−32.53, −8.72)	−0.06	0.001	0.03
DBP ^h^	3640	−6.68 (−12.80, −0.57)	−0.03	0.03	0.12
eGFR, mL/min × 1.73m^2 i^	3624	−3.08 (−10.80, 4.61)	−0.01	0.43	0.03
cIMT, mm ^j^	3625	0.04 (−0.03, 0.11)	0.02	0.27	0.05
CRP, mg/L ^k^	3629	−2.07 (−3.14, −1.37)	−0.06	0.001	0.07

^a^ Sex, smoking, alcohol consumption, DM, BMI, lipid lowering drugs. ^b^ Sex, smoking, alcohol consumption, DM, BMI, lipid lowering drugs. ^c^ Sex, smoking, alcohol consumption, DM, BMI, lipid lowering drugs. ^d^ Sex, smoking, alcohol consumption, BMI, glucose-lowering drugs. ^e^ Sex, smoking, alcohol consumption, BMI, glucose-lowering drugs. ^f^ Sex, smoking, alcohol consumption, DM, physical activity, higher education. ^g^ Sex, smoking, alcohol consumption, DM, BMI. ^h^ Sex, smoking, alcohol consumption, DM, BMI. ^i^ Sex, smoking, alcohol consumption, DM, BMI, hypertension. ^j^ Sex, smoking, alcohol consumption, DM, BMI, lipid lowering drugs, hypertension. ^k^ Sex, smoking, alcohol consumption, DM, BMI. Unstandardized β coefficients (Unstd. β-coeff.) with corresponding 95% confidence intervals (CI), standardized β coefficients (Std. β-coeff.), *p*-values and explained variance (R^2^) are given for plasma industrial trans fatty acid levels in univariable analysis and the fully adjusted multivariable models. Abbreviations: BMI: Body mass index. cIMT: Carotid intima-media thickness. CRP: C-reactive protein. DBP: Diastolic blood pressure. DM: Diabetes mellitus. eGFR: Estimated glomerular filtration rate. FPG: Fasting plasma glucose. HbA1c: Hemoglobin A1c. HDL: High-density lipoprotein. LDL: Low-density lipoprotein. SBP: Systolic blood pressure.

**Table 3:** Associations between plasma ruminant trans fatty acid levels and cardiovascular risk factors.

**Univariable Linear Regression Analysis**					
**Cardiovascular Risk Factors**	***n***	**Unstd. β-coeff. (95% CI)**	**Std. β-coeff.**	***p***	**R^2^**
HDL cholesterol, mmol/L ^a^	3680	0.25 (0.18, 0.32)	0.12	<0.001	0.01
LDL cholesterol, mmol/L ^b^	3657	−0.62 (−0.76, −0.49)	−0.15	<0.001	0.02
Triglycerides, mmol/L ^c^	3680	−1.51 (−1.61, −1.41)	−0.20	<0.001	0.04
FPG, mmol/L ^d^	3675	−1.11 (−1.14, −1.08)	−0.14	<0.001	0.02
HbA1c, % ^e^	3669	−1.08 (−1.10, −1.07)	−0.19	<0.001	0.04
BMI, kg/m^2 f^	3683	−2.60 (−3.21, −1.99)	−0.14	<0.001	0.02
SBP ^g^	3679	−5.14 (−7.75, −2.53)	−0.06	<0.001	0.004
DBP ^h^	3679	−3.40 (−4.80, −2.01)	−0.08	<0.001	0.01
eGFR, mL/min × 1.73m^2 i^	3664	−0.01 (−1.68, 1.66)	0.00	0.99	0.00
cIMT, mm ^j^	3661	−0.002 (−0.02, 0.01)	−0.004	0.83	0.00
CRP, mg/L ^k^	3669	−1.12 (−1.23, −1.02)	−0.04	0.01	0.001
**Multivariable Linear Regression Analysis**					
**Cardiovascular Risk Factors**	***n***	**Unstd. β-coeff. (95% CI)**	**Std. β-coeff.**	***p***	**R^2^**
HDL cholesterol, mmol/L ^a^	3640	0.16 (0.10, 0.22)	0.08	<0.001	0.27
LDL cholesterol, mmol/L ^b^	3617	−0.42 (−0.54, −0.30)	−0.10	<0.001	0.28
Triglycerides, mmol/L ^c^	3640	−1.37 (−1.46, −1.29)	−0.15	<0.001	0.21
FPG, mmol/L ^d^	3636	−1.06 (−1.08, −1.04)	−0.08	<0.001	0.39
HbA1c, % ^e^	3630	−1.06 (−1.07, −1.04)	−0.13	<0.001	0.39
BMI, kg/m^2 f^	3612	−1.96 (−2.55, −1.37)	−0.10	<0.001	0.10
SBP ^g^	3640	−3.31 (−5.92, −0.69)	−0.04	0.01	0.03
DBP ^g^	3640	−2.98 (−4.32, −1.64)	−0.07	<0.001	0.12
eGFR, mL/min × 1.73m^2 i^	3624	−0.83 (−2.52, 0.85)	−0.02	0.33	0.03
cIMT, mm ^j^	3625	0.01 (−0.01, 0.02)	0.01	0.56	0.05
CRP, mg/L ^k^	3629	−1.03 (−1.12, 1.04)	−0.01	0.49	0.07

^a^ Sex, smoking, alcohol consumption, DM, BMI, lipid lowering drugs. ^b^ Sex, smoking, alcohol consumption, DM, BMI, lipid lowering drugs. ^c^ Sex, smoking, alcohol consumption, DM, BMI, lipid lowering drugs. ^d^ Sex, smoking, alcohol consumption, BMI, glucose-lowering drugs. ^e^ Sex, smoking, alcohol consumption, BMI, glucose-lowering drugs. ^f^ Sex, smoking, alcohol consumption, DM, physical activity, higher education. ^g^ Sex, smoking, alcohol consumption, DM, BMI. ^h^ Sex, smoking, alcohol consumption, DM, BMI. ^i^ Sex, smoking, alcohol consumption, DM, BMI, hypertension. ^j^ Sex, smoking, alcohol consumption, DM, BMI, lipid lowering drugs, hypertension. ^k^ Sex, smoking, alcohol consumption, DM, BMI. Unstandardized β coefficients (Unstd. β-coeff.) with corresponding 95% confidence intervals (CI), standardized β coefficients (Std. β-coeff.), *p*-values and explained variance (R^2^) are given for plasma ruminant trans fatty acid levels in univariable analysis and the fully adjusted multivariable models. Abbreviations: BMI: Body mass index. cIMT: Carotid intima-media thickness. CRP: C-reactive protein. DBP: Diastolic blood pressure. DM: Diabetes mellitus. eGFR: Estimated glomerular filtration rate. FPG: Fasting plasma glucose. HbA1c: Hemoglobin A1c. HDL: High-density lipoprotein. LDL: Low-density lipoprotein. SBP: Systolic blood pressure.

## Supplementary Materials

Available online at https://www.mdpi.com/2072-6643/12/5/1419/s1, Table S1: Characteristics of study participants according to quartiles of ruminant trans fatty acid levels.

## References

[1] Micha R., Mozaffarian D. (2008). Trans fatty acids: Effects on cardiometabolic health and implications for policy. Prostaglandins Leukot. Essent. Fat. Acids.

[2] Mozaffarian D., Katan M.B., Ascherio A., Stampfer M.J., Willett W.C. (2006). Trans fatty acids and cardiovascular disease. N. Engl. J. Med..

[3] Willett W.C., Stampfer M.J., Manson J.E., Colditz G.A., Speizer F.E., Rosner B.A., Sampson L.A., Hennekens C.H. (1993). Intake of trans fatty acids and risk of coronary heart disease among women. Lancet.

[4] Ascherio A., Rimm E.B., Giovannucci E.L., Spiegelman D., Stampfer M., Willett W.C. (1996). Dietary fat and risk of coronary heart disease in men: Cohort follow up study in the United States. BMJ.

[5] Pietinen P., Ascherio A., Korhonen P., Hartman A.M., Willett W.C., Albanes D., Virtamo J. (1997). Intake of fatty acids and risk of coronary heart disease in a cohort of Finnish men. The Alpha-Tocopherol, Beta-Carotene Cancer Prevention Study. Am. J. Epidemiol..

[6] Oomen C.M., Ocke M.C., Feskens E.J., van Erp-Baart M.A., Kok F.J., Kromhout D. (2001). Association between trans fatty acid intake and 10-year risk of coronary heart disease in the Zutphen Elderly Study: A prospective population-based study. Lancet.

[7] Oh K., Hu F.B., Manson J.E., Stampfer M.J., Willett W.C. (2005). Dietary fat intake and risk of coronary heart disease in women: 20 years of follow-up of the nurses′ health study. Am. J. Epidemiol..

[8] Astrup A. (2006). The trans fatty acid story in Denmark. Athe. Suppl..

[9] Stender S. (2015). In equal amounts, the major ruminant trans fatty acid is as bad for LDL cholesterol as industrially produced trans fatty acids, but the latter are easier to remove from foods. Am. J. Clin. Nutr..

[10] (2003). Diet, Nutrition and the Prevention of Chronic Diseases.

[11] Johansson L., Borgejordet A., Pedersen J.I. (2006). Trans fatty acids in the Norwegian diet. Tidsskr. Nor. Laegeforen..

[12] Laake I., Pedersen J.I., Selmer R., Kirkhus B., Lindman A.S., Tverdal A., Veierod M.B. (2012). A prospective study of intake of trans-fatty acids from ruminant fat, partially hydrogenated vegetable oils, and marine oils and mortality from CVD. Br. J. Nutr..

[13] Gebauer S.K., Destaillats F., Dionisi F., Krauss R.M., Baer D.J. (2015). Vaccenic acid and trans fatty acid isomers from partially hydrogenated oil both adversely affect LDL cholesterol: A double-blind, randomized controlled trial. Am. J. Clin. Nutr..

[14] Baylin A., Kim M.K., Donovan-Palmer A., Siles X., Dougherty L., Tocco P., Campos H. (2005). Fasting whole blood as a biomarker of essential fatty acid intake in epidemiologic studies: Comparison with adipose tissue and plasma. Am. J. Epidemiol..

[15] Berge T., Vigen T., Pervez M.O., Ihle-Hansen H., Lyngbakken M.N., Omland T., Smith P., Steine K., Rosjo H., Tveit A. (2015). Heart and Brain Interactions—The Akershus Cardiac Examination (ACE) 1950 Study Design. Scand. Cardiovasc. J..

[16] Svilaas A., Strom E.C., Svilaas T., Borgejordet A., Thoresen M., Ose L. (2002). Reproducibility and validity of a short food questionnaire for the assessment of dietary habits. Nutr. Metab. Cardiovasc. Dis..

[17] Morseth B., Hopstock L.A. (2020). Time trends in physical activity in the Tromso study: An update. PLoS ONE.

[18] Lamprea-Montealegre J.A., Zelnick L.R., Hall Y.N., Bansal N., de Boer I.H. (2018). Prevalence of Hypertension and Cardiovascular Risk According to Blood Pressure Thresholds Used for Diagnosis. Hypertension.

[19] (2000). Obesity: Preventing and Managing the Global Epidemic: Report of a WHO Consultation.

[20] Expert Panel on Detection E (2001). Treatment of High Blood Cholesterol in A: Executive Summary of The Third Report of The National Cholesterol Education Program (NCEP) Expert Panel on Detection, Evaluation, And Treatment of High Blood Cholesterol in Adults (Adult Treatment Panel III). JAMA.

[21] American Diabetes Association (2014). Diagnosis and Classification of Diabetes Mellitus. Diabetes Care.

[22] Levey A.S., Stevens L.A., Schmid C.H., Zhang Y.L., Castro A.F., Feldman H.I., Kusek J.W., Eggers P., Van Lente F., Greene T. (2009). A new equation to estimate glomerular filtration rate. Ann. Intern. Med..

[23] Ihle-Hansen H., Vigen T., Ihle-Hansen H., Ronning O.M., Berge T., Thommessen B., Lyngbakken M.N., Orstad E.B., Enger S., Nygard S. (2018). Prevalence of Carotid Plaque in a 63- to 65-Year-Old Norwegian Cohort From the General Population: The ACE (Akershus Cardiac Examination) 1950 Study. J. Am. Heart Assoc..

[24] Eide I.A., Jenssen T., Hartmann A., Diep L.M., Dahle D.O., Reisaeter A.V., Bjerve K.S., Christensen J.H., Schmidt E.B., Svensson M. (2015). The association between marine n-3 polyunsaturated fatty acid levels and survival after renal transplantation. Clin. J. Am. Soc. Nephrol..

[25] Gebauer S.K., Chardigny J.M., Jakobsen M.U., Lamarche B., Lock A.L., Proctor S.D., Baer D.J. (2011). Effects of ruminant trans fatty acids on cardiovascular disease and cancer: A comprehensive review of epidemiological, clinical, and mechanistic studies. Adv. Nutr..

[26] Mensink R.P., Zock P.L., Kester A.D., Katan M.B. (2003). Effects of dietary fatty acids and carbohydrates on the ratio of serum total to HDL cholesterol and on serum lipids and apolipoproteins: A meta-analysis of 60 controlled trials. Am. J. Clin. Nutr..

[27] Mozaffarian D. (2006). Trans fatty acids—Effects on systemic inflammation and endothelial function. Atheroscler. Suppl..

[28] Tardy A.L., Morio B., Chardigny J.M., Malpuech-Brugere C. (2011). Ruminant and industrial sources of trans-fat and cardiovascular and diabetic diseases. Nutr. Res. Rev..

[29] Wilczek M.M., Olszewski R., Krupienicz A. (2017). Trans-Fatty Acids and Cardiovascular Disease: Urgent Need for Legislation. Cardiology.

[30] Ascherio A., Hennekens C.H., Buring J.E., Master C., Stampfer M.J., Willett W.C. (1994). Trans-fatty acids intake and risk of myocardial infarction. Circulation.

[31] Stender S., Astrup A., Dyerberg J. (2016). Artificial trans fat in popular foods in 2012 and in 2014: A market basket investigation in six European countries. BMJ Open.

[32] Ministers N.C. (2014). Nordic Nutrition Recommendations 2012. https://www.nordic-ilibrary.org/content/publication/nord2014-002.

[33] Chandra A., Svensson M., Asberg A., Schmidt E.B., Bjerve K.S., Jenssen T., Hartmann A., Ueland T., Eide I.A. (2019). Trans-fatty Acids and Survival in Renal Transplantation. J. Ren. Nutr..

[34] Lefevre M., Lovejoy J.C., Smith S.R., Delany J.P., Champagne C., Most M.M., Denkins Y., de Jonge L., Rood J., Bray G.A. (2005). Comparison of the acute response to meals enriched with cis- or trans-fatty acids on glucose and lipids in overweight individuals with differing FABP2 genotypes. Metabolism.

[35] Field A.E., Willett W.C., Lissner L., Colditz G.A. (2007). Dietary fat and weight gain among women in the Nurses’ Health Study. Obesity (Silver Spring).

[36] Wang L., Manson J.E., Forman J.P., Gaziano J.M., Buring J.E., Sesso H.D. (2010). Dietary fatty acids and the risk of hypertension in middle-aged and older women. Hypertension.

[37] Mozaffarian D., Pischon T., Hankinson S.E., Rifai N., Joshipura K., Willett W.C., Rimm E.B. (2004). Dietary intake of trans fatty acids and systemic inflammation in women. Am. J. Clin. Nutr..

[38] Transfettsyrer. https://www.matportalen.no/kosthold_og_helse/transfettsyrer.

[39] Stender S., Astrup A., Dyerberg J. (2008). Ruminant and industrially produced trans fatty acids: Health aspects. Food Nutr Res..

[40] Jakobsen M.U., Bysted A., Andersen N.L., Heitmann B.L., Hartkopp H.B., Leth T., Overvad K., Dyerberg J. (2006). Intake of ruminant trans fatty acids in the Danish population aged 1–80 years. Eur. J. Clin. Nutr..

[41] Motard-Belanger A., Charest A., Grenier G., Paquin P., Chouinard Y., Lemieux S., Couture P., Lamarche B. (2008). Study of the effect of trans fatty acids from ruminants on blood lipids and other risk factors for cardiovascular disease. Am. J. Clin. Nutr..

[42] Mozaffarian D., Cao H., King I.B., Lemaitre R.N., Song X., Siscovick D.S., Hotamisligil G.S. (2010). Trans-palmitoleic acid, metabolic risk factors, and new-onset diabetes in U.S. adults: A cohort study. Ann. Intern. Med..

[43] Mozaffarian D., de Oliveira Otto M.C., Lemaitre R.N., Fretts A.M., Hotamisligil G., Tsai M.Y., Siscovick D.S., Nettleton J.A. (2013). Trans-Palmitoleic acid, other dairy fat biomarkers, and incident diabetes: The Multi-Ethnic Study of Atherosclerosis (MESA). Am. J. Clin. Nutr..

[44] Tholstrup T., Raff M., Basu S., Nonboe P., Sejrsen K., Straarup E.M. (2006). Effects of butter high in ruminant trans and monounsaturated fatty acids on lipoproteins, incorporation of fatty acids into lipid classes, plasma C-reactive protein, oxidative stress, hemostatic variables, and insulin in healthy young men. Am. J. Clin. Nutr..

[45] Hansen C.P., Berentzen T.L., Halkjær J., Tjønneland A., Sørensen T.I., Overvad K., Jakobsen M.U. (2012). Intake of ruminant trans fatty acids and changes in body weight and waist circumference. Eur. J. Clin. Nutr..

[46] Hansen C.P., Heitmann B.L., Sørensen TIa Overvad K., Jakobsen M.U. (2016). Intake of ruminant trans-fatty acids, assessed by diet history interview, and changes in measured body size, shape and composition. Public Health Nutr..

[47] Mensink R.P., de Louw M.H., Katan M.B. (1991). Effects of dietary trans fatty acids on blood pressure in normotensive subjects. Eur. J. Clin. Nutr..

[48] Brouwer I.A., Wanders A.J., Katan M.B. (2013). Trans fatty acids and cardiovascular health: Research completed?. Eur. J. Clin. Nutr..

[49] Micha R., Khatibzadeh S., Shi P., Fahimi S., Lim S., Andrews K.G., Engell R.E., Powles J., Ezzati M., Mozaffarian D. (2014). Global, regional, and national consumption levels of dietary fats and oils in 1990 and 2010: A systematic analysis including 266 country-specific nutrition surveys. BMJ.

[50] Wanders A.J., Zock P.L., Brouwer I.A. (2017). Trans Fat Intake and Its Dietary Sources in General Populations Worldwide: A Systematic Review. Nutrients.

[51] Restrepo B.J., Rieger M. (2016). Denmark′s Policy on Artificial Trans Fat and Cardiovascular Disease. Am. J. Prev. Med..

