# Supplementary material for: Plasma Trans Fatty Acid Levels, Cardiovascular Risk Factors and Lifestyle: Results from the Akershus Cardiac Examination 1950 Study
Source: Nutrients. 2020 May 14;12(5):1419. doi: 10.3390/nu12051419 (PMC7284775; doi:10.3390/nu12051419)
Supplement: Supplementary file 1 [file nutrients-12-01419-s001.pdf]

Supplementary Table 1. Characteristics of study participants according to quartiles of ruminant trans fatty acid levels.

	All participants	Quartile 1	Quartile 2	Quartile 3	Quartile 4	p for trend	p for Q1 vs. Q4
**Ruminant trans fatty acid level, *wt%***	**0.14-2.87**	**≤1.45**	**1.46–1.60**	**1.61-1.75**	**≥1.76**		
Number of participants	3683	900	995	874	914		
Age, *years*	63.9 (0.6)	63.8 (0.6)	63.9 (0.6)	64.0 (0.6)	64.0 (0.6)	<0.001	<0.001
Gender (Male), %	51.3 (n=1890)	51.8 (n=466)	49.8 (n=496)	51.7 (n=452)	52.1 (n=476)	0.75	0.90
Weekly intake of milk, 1 glass or more, %	56.5 (n=2042)	56.2 (n=491)	57.9 (n=566)	55.6 (n=480)	56.2 (n=505)	0.76	0.99
Use butter or margarine in cooking, %	50.4 (n=1782)	47.8 (n=407)	49.5 (n=474)	51.5 (n=435)	52.6 (n=466)	0.19	0.04
Use oil in cooking, %	48.2 (n=1706)	50.9 (n=434)	48.9 (n=468)	47.5 (n=401)	45.5 (n=403)	0.14	0.02
Weekly intake of fast food, once or more, %	18.1 (n=651)	17.0 (n=148)	17.5 (n=170)	20.7 (n=178)	17.2 (n=155)	0.15	0.91
Current smoker, %	14.5 (n=530)	12.9 (n=115)	12.4 (n=122)	16.0 (n=139)	17.0 (n=154)	0.01	0.02
Alcohol consumption, (≥ 2 times weekly), %	47.4 (n=1739)	43.5 (n=390)	45.9 (n=456)	48.7 (n=425)	51.5 (n=468)	0.01	0.001
Alcohol consumption, > 5 units at same occasion, %	46.6 (n=1701)	40.0 (n=354)	42.7 (n=422)	50.6 (n=440)	53.7 (n=485)	<0.001	<0.001
Physical activity (≥ 2 times weekly), %	61.7 (n=2240)	59.2 (n=520)	63.6 (n=620)	62.8 (n=544)	61.6 (n=556)	0.27	0.30
Higher education, %	46.5	45.2	46.8	49.5	44.6	0.17	0.80

	(n=1708)	(n=405)	(n=465)	(n=432)	(n=406)		
Hypertension, %	62.0 (n=2282)	63.9 (n=575)	62.1 (n=617)	60.2 (n=526)	61.7 (n=564)	0.45	0.34
Hypercholesterolemia, %	52.6 (n=1931)	52.2 (n=468)	51.2 (n=508)	51.7 (n=450)	55.3 (n=505)	0.27	0.18
Cerebrovascular disease, %	3.7 (n=138)	3.6 (n=32)	3.3 (n=33)	3.5 (n=31)	4.6 (n=42)	0.47	0.26
Coronary artery disease, %	7.0 (n=259)	5.1 (n=46)	6.5 (n=65)	6.6 (n=58)	9.8 (n=90)	0.001	<0.001
Diabetes mellitus, %	8.5 (n=313)	12.2 (n=110)	9.8 (n=98)	6.3 (n=55)	5.5 (n=50)	<0.001	<0.001
Obesity (BMI ≥ 30), %	22.6 (n=831)	29.7 (n=267)	22.9 (n=228)	19.3 (n=169)	18.3 (n=167)	<0.001	<0.001
CKD stages 3-5 (eGFR <60 ml/min x 1.73m^2), %	3.9 (n=142)	3.2 (n=29)	3.7 (n=37)	3.8 (n=33)	4.7 (n=43)	0.42	0.10
Medication, %							
Diuretics	3.1	4.0	2.4	3.5	2.4	0.11	0.05
Beta blockers	13.4	13.0	12.3	13.6	14.7	0.47	0.31
Calcium channel blockers	8.1	8.1	9.1	8.1	7.1	0.45	0.99
ACEi or ARB	26.9	29.4	26.0	26.0	26.4	0.28	0.14
Lipid-lowering drugs	26.1	22.3	23.9	25.9	32.6	<0.001	<0.001
Glucose-lowering drugs	5.4	7.8	6.1	4.2	3.4	<0.001	<0.001
Systolic blood pressure, *mmHg*	138 (19)	139 (18)	139 (19)	137 (19)	136 (20)	0.003	0.001
Diastolic blood pressure, *mmHg*	77 (10)	78 (10)	77 (10)	77 (10)	76 (10)	<0.001	<0.001

HDL cholesterol, *mmol/L*	1.5 (0.5)	1.5 (0.5)	1.5 (0.5)	1.5 (0.5)	1.6 (0.5)	<0.001	<0.001
LDL cholesterol, *mmol/L*	3.3 (1.0)	3.4 (1.0)	3.4 (1.0)	3.3 (0.9)	3.1 (1.0)	<0.001	<0.001
Triglycerides, *mmol/L*	1.2 (0.4-2-0)	1.4 (0.4-2.4)	1.2 (0.4-2.0)	1.1 (0.3-1.9)	1.0 (0.4-1.6)	<0.001	<0.001
Fasting plasma glucose, *mmol/L*	5.3 (4.5-6.1)	5.4 (4.5-6.3)	5.3 (4.5-6.1)	5.3 (4.5-6.1)	5.2 (4.5-5.9)	<0.001	<0.001
HbA1c, %	5.7 (5.3-6.2)	5.8 (5.3-6.3)	5.7 (5.3-6.1)	5.7 (5.3-6.1)	5.6 (5.2-6.0)	<0.001	<0.001
Body mass index (BMI), *kg/m*^{2}	27.1 (4.4)	28.0 (4.4)	27.4 (4.4)	26.9 (4.2)	26.4 (4.5)	<0.001	<0.001
eGFR, *ml/min x 1.73m2*	83 (11.9)	84 (11.6)	83 (12.2)	83 (12.0)	83 (11.8)	0.14	0.50
cIMT, *mm*	0.73 (0.1)	0.73 (0.1)	0.73 (0.1)	0.73 (0.1)	0.73 (0.1)	0.87	0.64
CRP, *mg/L*	1.5 (1.4-1.6)	1.5 (1.4-1.6)	1.5 (1.4-1.6)	1.5 (1.4-1.6)	1.5 (1.4-1.6)	-	-
Marine n-3 PUFAs, *wt%*	8.1 (2.6)	7.5 (2.4)	8.1 (2.5)	8.3 (2.7)	8.3 (2.8)	<0.001	<0.001
LA, *wt%*	20.8 (3.0)	21.5 (3.0)	21.1 (2.8)	20.6 (2.9)	20.0 (2.9)	<0.001	<0.001
AA, *wt%*	9.2 (2.1)	9.0 (1.9)	9.1 (1.9)	9.3 (2.0)	9.6 (2.3)	<0.001	<0.001
SFA, *wt%*	42.4 (0.9)	42.8 (0.8)	42.5 (0.8)	42.3 (0.8)	42.0 (0.9)	<0.001	<0.001
MUFA, *wt%*	10.3 (1.4)	10.1 (1.5)	10.1 (1.2)	10.3 (1.3)	10.6 (1.5)	<0.001	<0.001

Results are presented as percentage for categorical data, mean value (standard deviation) for continuous data and median value (inter-quartile ranges) for skewed data. Differences between groups were evaluated using Chi square for dichotomous data, the Kruskal-Wallis test for triglycerides, fasting plasma glucose, HbA1c and CRP, and ANOVA for other continuous data. Fatty acids are given as weight percentage (wt%) of total plasma phospholipid fatty acids. Abbreviations: BMI: Body mass index. CKD: Chronic kidney disease. eGFR: Estimated glomerular filtration rate (CKD-EPI formula). ACEi: Angiotensin converting enzyme inhibitor. ARB: Angiotensin receptor blocker. HDL: High density lipoprotein. LDL: Low density lipoproteins. HbA1c: Hemoglobin A1c. cIMT:

Carotid intima-media thickness. CRP: C-reactive protein. PUFA: Polyunsaturated fatty acids. LA: Linoleic acid. AA: Arachidonic acid. SFA: Saturated fatty acids. MUFA: Monounsaturated fatty acids.
